# Effects of Solution Treatment on Damping Capacities of Binary Mg-X (X = Ga and Er) Alloys

**DOI:** 10.3390/ma15228172

**Published:** 2022-11-17

**Authors:** Xiaoyang Dong, Jinxing Wang, Zichuan Wang, Xiyu Wang, Cong Dang, Zhicheng Wan, Yi Zou, Jingfeng Wang, Fusheng Pan

**Affiliations:** 1 College of Materials Science and Engineering, Chongqing University, Chongqing 400030, China; 2 National Engineering Research Center for Magnesium Alloys, Chongqing University, Chongqing 400030, China

**Keywords:** binary Mg-X (X = Ga and Er) alloys, solution treatment, damping capacity, damping mechanism

## Abstract

Designing new materials for vibration and noise reduction that are lightweight is of great significance for industrial development. Magnesium (Mg) alloy is considered one of the best damping metal structural materials because of its low density, high specific strength, good energy storage characteristics and rich resources. Solution atoms have an important effect on the damping capacities of Mg alloys, but the relevant laws have not been completely clarified. In this work, two kinds of alloying elements (Ga and Er) with various atomic sizes were selected to study the metallographic structure and damping capacities of binary Mg-X (X = Ga and Er) alloys in the as-cast and solid solution states, respectively. Solution treatment can improve the damping capacities of binary Mg-X (X = Ga and Er) alloys, and the damping mechanisms of the two solid solution alloys are consistent with the G-L damping mechanism. The influence of alloy elements with different atomic sizes on damping capacities is also different. This influence is due to the various radii of solute atoms and Mg atoms which can result in different degrees of lattice distortion. This work provides a research basis for development and design of high-performance damping Mg alloy materials.

## 1. Introduction

With the sustainable development of modern industry, the number of tools and high-power machinery that generate severe vibration is increasing. The vibration hazards brought by various mechanical equipment in operation and working process are also increasingly serious. In order to reduce the impact of such vibration and noise on people’s lives and work, modern instruments and equipment put forward more urgent requirements for damping noise reduction and making equipment lightweight [1,2,3]. Using damping materials to reduce vibration and noise has become one of the effective means to solve the above problems. Pure magnesium (Mg) has a special close-packed hexagonal crystal structure and the advantages of being lightweight, having high specific strength, superior damping and vibration reduction, excellent electromagnetic shielding performance and rich resources [4,5]. Among various structural metal materials, the damping capacity of Mg alloy is the best, making it a prominent candidate for the increasing demand for vibration and noise control in modern industry [6,7,8]. However, its comprehensive mechanical properties are poor and far from meeting the requirements of general structural materials [9]. Therefore, the design of lightweight and structural functional integration of metal damping materials will be the focus of future development.

Binary Mg alloy is an alloy composed of Mg as the matrix and other elements [10,11]. It owns the advantages of lower density, high specific strength, excellent energy storage characteristics, strong resistance to dynamic impact load, outstanding electromagnetic radiation shielding capability and abundant resources [12,13,14,15]. It is one of the metal structural materials with superior damping capacity at present, and has been widely used in aerospace, rail transit, national defense and military industry, automobile, electronic information and other fields [15,16]. According to G-L theory, high damping Mg alloys require good mobility of dislocations. Thus, there are three criteria for preparing Mg alloys with high damping capacities: (i) the content of solid solution elements in Mg alloys should be as small as possible; (ii) the grain size of Mg alloy should be larger than 10 μm; (iii) the second phase in Mg alloy is preferably dendritic structure with stress concentration effect [17,18,19,20,21]. Solution treatment can regulate the redistribution of the shape, size, number of the second phase or dislocation density in the material. This redistribution will affect the movement of dislocations, and then affect the damping capacity of the alloy [10,22,23,24,25]. Zhang et al. found that solution treatment made the Mg_17_Al_12_ phase form during solidification and dissolved and weakened the strong pinning points on dislocations, and increased the internal friction and damping capacity of AZ91D alloy [26]. Ren et al. found that the damping capacity of Mg-1 wt.% Y alloy in solution state was better than that of as-cast alloy [27]. Yu et al. explained the effects of four solute atoms on the damping properties of binary Mg alloys [22]. Researchers indicate that various solid solution elements have different effects on damping capacities of Mg alloys. However, the effect of solution treatment on the damping properties of Mg alloys has not been completely clarified.

In this work, two alloying elements (Ga and Er) with various atomic sizes were selected to study the metallographic structure and damping capacities of binary Mg-X (X = Ga and Er) alloys in the as-cast and solid solution states, respectively. The metallographic structure, microstructure and damping capacities of binary Mg-X (X = Ga and Er) alloys in the as-cast and solid solution states were observed and analyzed by characterization tests. The difference in damping capacity between as-cast and solid solution alloys in the same system was studied. The influence of different solute atoms on the damping capacity of the binary alloy was discussed. The damping mechanism of the binary Mg alloy in the solution state was also explored. This work investigates the effect of solution treatment on the damping properties and provides a basis for the preparation of new high-performance damping binary Mg alloys.

## 2. Materials and Methods

In this work, Ga and Er elements were selected to study the metallographic structure and damping capacities of binary Mg-X (X = Ga and Er) alloys in the as-cast and solid solution states, respectively. The atomic sizes of Mg, Ga, and Er were displayed in Table 1. The binary Mg-X (X = Ga and Er) alloys were melted in a vacuum induction melting furnace with Mg-5Ga and Mg-30Er master alloys as raw materials. The content of alloy elements was set to 1.6 at.% (at.% is the limiting solid solubility of atomic ratio). The value is far lower than the limiting solid solubility of alloy elements (Ga and Er) in Mg and can ensure that the second phase particles can be completely dissolved into the matrix after solution treatment. Before melting, the furnace was washed with pure Mg (99.95A, Mg ingot), and then the raw materials were put into the tungsten crucible according to the predetermined composition for melting. After ensuring the alloy homogenization, the alloys were poured into the stainless steel mold with a diameter of 50 mm. The sample was removed from the furnace when the furnace body was cooled to room temperature. The alloys were solution treated with an electric heating box furnace. The temperature of solution treatment is determined according to the solid solubility of binary Mg-X (X = Ga and Er) in the phase diagrams of Mg-Ga and Mg-Er alloys. The solution treatment cycles of Mg-Ga and Mg-Er alloys are 380 °C × 24 h and 540 °C × 24 h, respectively. The alloy sample was cut into rounds with a thickness of 10 × 10 × 5 mm. The etching solution consisted of ethanol (14 mL), acetic acid (10 mL) and picric acid (3 g). The above mixture was used to etch the surface of the alloy samples.

The microstructure of binary Mg-X (X = Ga and Er) alloys in as-cast and solid solution states were observed by optical microscope (OM). The phase constitutions of the alloys were analyzed by a Rigaku D/max 2500PC X-ray diffraction (XRD) technique at 40 kV and 150 mA with Cu Kα radiation at a scanning rate of 4°/min from 10° to 90° and a step size of 0.02°. The microstructure and phase composition of the alloys were analyzed with a JSM-7800F scanning electron microscope (SEM) and by energy dispersive spectroscopy (EDS). The tensile property measurements were carried out on the CMT-5105 electronic universal testing machine. The tensile sample size is 12.5 × 5.0 × 2.0 mm and the strain rate is 1 mm/min. The damping capacity of alloys was investigated by a dynamic mechanical thermal analyzer (DMA850) which adopts a single cantilever clamp and forced vibration mode. The damping tests were conducted at room temperature. The test frequency was set as 1.0 Hz. The strain amplitude (ε) was ranged from 1 × 10^−5^ to 2 × 10^−3^. The size of the prepared damping test samples is 40.0 × 5.0 × 2.0 mm. The damping capacities of alloys were assessed with Q^−1^ (Q^−1^ = tan *Φ*, where *Φ* represents the lag angle between the applied strain and response stress) in this work [22]. To ensure the accuracy of the experimental data, three parallel samples were taken for each alloy to be tested.

## 3. Results and Discussion

### 3.1. Microstructures of Binary Mg-X (X = Ga and Er) Alloys

**Figure 1:**
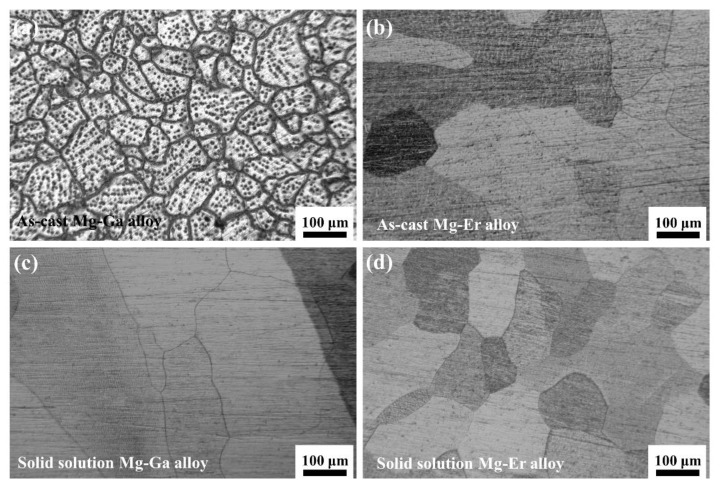
OM images of (**a**) as-cast Mg-Ga alloy; (**b**) as-cast Mg-Er alloy; (**c**) solid solution Mg-Ga alloy; (**d**) solid solution Mg-Er alloy.

The OM images of four binary Mg-X (X = Ga and Er) alloys are displayed in Figure 1. Figure 1a shows the microstructure of the as-cast Mg-Ga alloy. The grains show a reticular distribution with small grain size and many black second phases exist in the matrix. The high content of Ga element added can reduce the tension and nucleation energy at the interface between solid and liquid phases and the critical nucleation radius, playing a role in grain refinement [28,29]. Figure 1b represents the microstructure of as-cast Mg-Er alloy. The grains are distributed in a network with large grain size and some black second phases can be observed in the matrix. Figure 1c reveals the microstructure of the solid solution Mg-Ga alloy. Compared with the as-cast Mg-Ga alloy, the second phase basically disappears, and Ga elements exist in the matrix of solid solution Mg-Ga alloy in the form of solid solution atoms. Through the optimization of the solution treatment process, the grain of the solution state Mg-Ga alloy is uniform, and the average size is about 280 μm. Figure 1d exhibits the microstructure of the solid solution Mg-Er alloy. Compared with the as-cast Mg-Er alloy, the second phase basically disappears in the matrix of solid solution Mg-Er alloy. After solid solution treatment, the grains of the Mg-Er alloy are equiaxed and uniform. The average grain size of solid solution Mg-Er alloy is about 260 μm. After solution treatment, the microstructure of binary Mg-X (X = Ga and Er) alloys is basically composed of α-Mg grain composition, and almost no grain boundary compounds are observed. However, most of the grains are equiaxed and the grain size is uniform [23].

**Figure 2:**
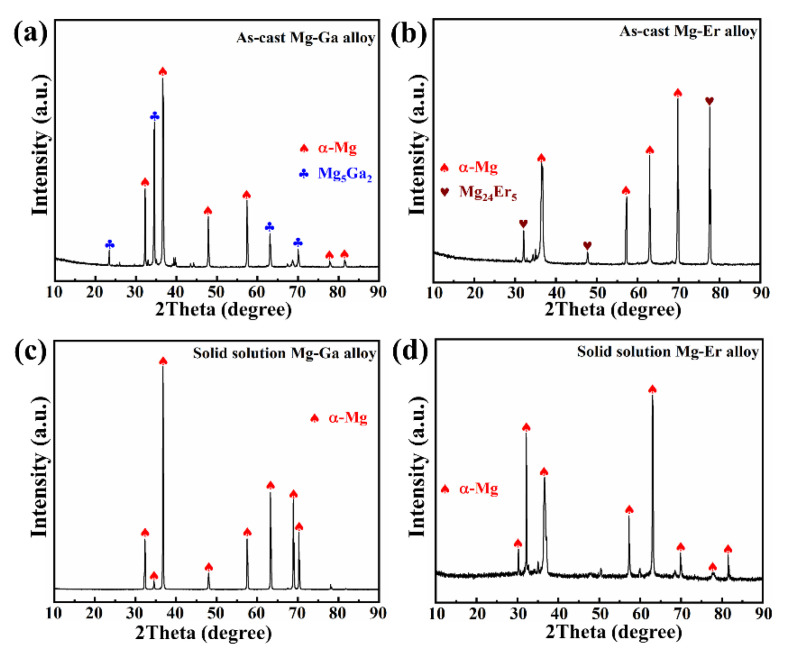
XRD patterns of (**a**) as-cast Mg-Ga alloy; (**b**) as-cast Mg-Er alloy; (**c**) solid solution Mg-Ga alloy; (**d**) solid solution Mg-Er alloy.

Figure 2 reveals the XRD patterns of four binary Mg-X (X = Ga and Er) alloys. Figure 2a shows the XRD pattern of as-cast Mg-Ga alloy, composed of α-Mg matrix phase and Mg_5_Ga_2_ phase. Figure 2b displays the XRD pattern of as-cast Mg-Er alloy, consisting of α-Mg matrix phase and Mg_24_Er_5_ phase. Figure 2c represents the XRD pattern of the solid solution Mg-Ga alloy. It can be found that only α-Mg matrix phase exists in the alloy, suggesting that the second phase in the alloy is dissolved after solution treatment and Ga element exists in the form of solute atom [30]. Figure 2d shows the XRD pattern of the solid solution Mg-Er alloy. Only the α-Mg phase exists in the matrix of solid solution Mg-Er alloy, indicating that the second phase in the alloy is dissolved and the Er element exists in the form of solute atoms [31]. The above results suggest that the second phase in the alloy will dissolve after solution treatment and the alloy elements exist in the form of solute atoms. The diffraction peaks of the two solid solution Mg-X (X = Ga and Er) alloys shift to higher angles [32,33]. When the radius difference between Mg and solute atoms is larger, the displacement of the diffraction peaks is larger [22].

**Figure 3:**
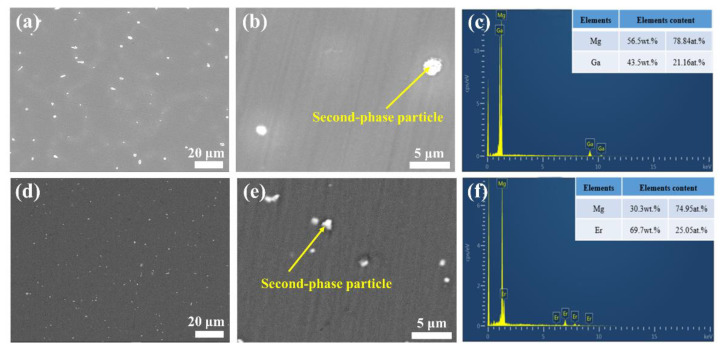
(**a**,**b**) SEM images of as-cast Mg-Ga alloy; (**c**) the corresponding EDS result of as-cast Mg-Ga alloy; (**d**,**e**) SEM images of as-cast Mg-Er alloy; (**f**) the corresponding EDS result of as-cast Mg-Er alloy.

The existence of the second phase can be observed in the binary Mg-X (X = Ga and Er) as-cast alloys. The microstructure and composition of two alloys were observed and analyzed by SEM and EDS, respectively. The corresponding SEM and EDS results of two as-cast alloys are exhibited in Figure 3. As shown in Figure 3a, the as-cast Mg-Ga alloy has many bright white particles distributed on the alloy matrix. Figure 3b,c shows the SEM image of as-cast Mg-Ga alloy and corresponding EDS results of the white particles. According to the analysis of the atomic percentage results of EDS, the white particles are composed of Mg and Ga elements and belong to the second phase of Mg_5_Ga_2_. As shown in Figure 3d, the as-cast Mg-Er alloy also has many bright white particles distributed on the alloy matrix. The morphology of the second phase and the EDS results of the selected area are shown in Figure 3e,f. According to the EDS results, the white particles is the second phase and composed of Mg and Er elements. The second phase is Mg_24_Er_5_ phase depending on the EDS atomic percentage analysis. This result is consistent with the previous XRD analysis, indicating that the second phase does exist in the as-cast alloy.

According to the OM images, XRD analysis, SEM and EDS results, the second phase exists in the binary Mg-X (X = Ga and Er) as-cast alloys dispersed in the Mg matrix, indicating that the alloy elements exist in the form of the second phase in the as-cast alloy [29,31]. However, no second phase was observed in the grain interior and along the grain boundary of the solid solution alloys, indicating that the alloy elements in the solid solution alloy were completely dissolved and uniformly distributed in the matrix.

### 3.2. Effects of Solution Treatment on Damping Capacity

**Figure 4:**
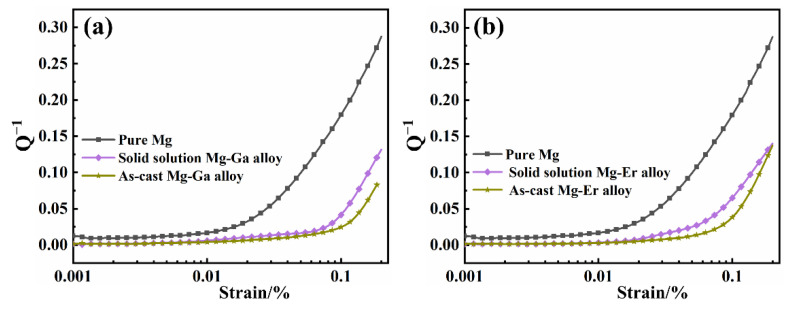
(**a**) The damping curves of as-cast, solid solution Mg-Ga alloy and pure Mg; (**b**) the damping curves of as-cast, solid solution Mg-Er alloy and pure Mg.

The damping capacities of the four binary Mg-X (X = Ga and Er) alloys are shown in Figure 4. Figure 4a shows the strain damping curve of as-cast, solid solution Mg-Ga alloy and pure Mg. In the whole strain stage, the damping capacity of as-cast Mg-Ga alloy is less than that of solid solution Mg-Ga alloy and pure Mg. When the strain arrives at 10^−3^, the damping values of as-cast and solid solution Mg-Ga alloy are 0.024 and 0.042, respectively. Figure 4b represents the strain damping curve of as-cast, solid solution Mg-Er alloy and pure Mg. When the strain is 10^−3^, the damping values of as-cast and solid solution Mg-Er alloy are 0.038 and 0.065, respectively. The damping values of four binary Mg-X (X = Ga and Er) alloys and pure Mg are shown in Table 2. According to the strain damping curve, the damping capacity of Mg-X (X = Ga and Er) alloys is weakly affected by the change of strain at the low strain stage. However, the damping capacity of Mg-X (X = Ga and Er) alloys has been greatly improved at the high strain stage, indicating that there is a certain correlation between the damping property of alloys and the strain amplitude [8,12]. In the low strain stage, the stress of binary alloy is small, and the dislocation is pinned by the weak nail rolling point and cannot slip. The dislocation can only make a reciprocating bow movement between the weak nail rolling points, thus consuming energy and generating damping that is independent of the strain amplitude and frequency. In the high strain stage, the dislocation will be unpinned from the weak pinning point with the increase in stress, but these dislocations will continue to be pinned by the grain boundary strong nail rolling point. As the stress continues to increase, the area swept by the dislocation section under the action of external force increases, the vibration energy dissipation of the material increases, and the damping property of the alloys increases sharply [13,15]. In addition, compared with binary Mg-X (X = Ga and Er) as-cast alloys, the damping properties of binary Mg-X (X = Ga and Er) solid solution alloys are improved after solution treatment. This is because the solution treatment reduces the content of the second phase and the number of strong pinning points, and increases the average dislocation movable length of the movable dislocation line between the strong pinning points, improving the damping capacity of alloy. At the same time, the alloy elements can reduce the difference of critical shear stress between non-basal plane and basal plane, and improve the mobility of dislocations [22,23,27]. Therefore, the damping capacity of alloy is improved after solution treatment.

### 3.3. Effects of Solute Atoms on Damping Capacity

**Figure 5:**
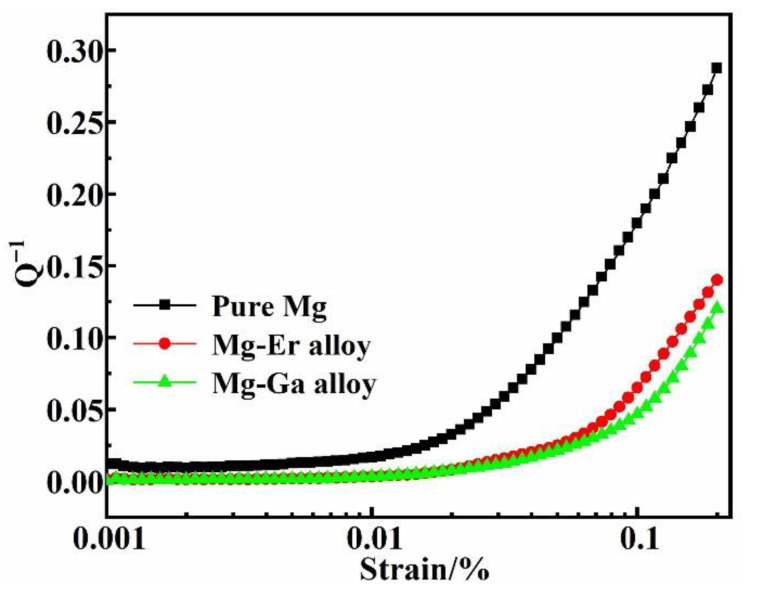
The damping curves of pure Mg, solid solution Mg-Ga and Mg-Er alloys.

In the two Mg-X (X = Ga and Er) solid solution alloys, the alloy elements of Ga and Er exist in the Mg matrix in the form of solute atoms. Due to the different atomic characteristics of the alloy elements, such as atomic radius, electronegativity and mixing enthalpy, Ga and Er produce different lattice distortion and binding force in the Mg matrix and have different hindering effects on dislocation movement, and the alloys exhibit different damping capacities [10]. As can be seen from Table 3, the difference of atomic radius between the alloy elements Er and Mg is small, while the difference of atomic radius between the alloy elements Ga and Mg is large. Figure 5 displays the strain damping curves of solid solution Mg-Ga and Mg-Er alloys. When the solute atom dissolves into the matrix, due to the difference in atomic radius and elastic modulus between the solute atom and Mg, the original regular lattice will be distorted to varying degrees, affecting the dislocation movement, and thus affecting the mechanical properties and damping properties of the alloys. Therefore, the lattice distortion caused by the dissolution of Er into the Mg matrix is small, while the lattice distortion caused by the dissolution of Ga into the Mg matrix is large. The smaller the lattice distortion is, the smaller the critical shear stress is required for dislocation movement. The dislocation movement can consume energy and contribute to better damping performance [22]. When the grain size of alloys is similar and the content of alloy elements is similar, the damping properties of solid solution alloys are different, and the difference of atomic radius is the dominant factor.

### 3.4. Damping Mechanism of Binary Solid Solution Mg-X (X = Ga and Er) Alloys

The high-performance damping in Mg alloys is mainly due to the dislocation damping. The corresponding damping capacity could be clarified by the G-L theory to explain the effect of alloying elements on the dislocation of Mg alloys. Depending on this model, the strain dependence of damping capacity can be revealed in two stages and the equations can be obtained as follows [18,19,20,22]:

Q^−1^(ε) = Q_o_^−1^ + Q_H_^−1^(ε) (1)

(2) $${Q_{o}}^{-1}\sim \rho L_{C}^{4}$$

**Figure 6:**
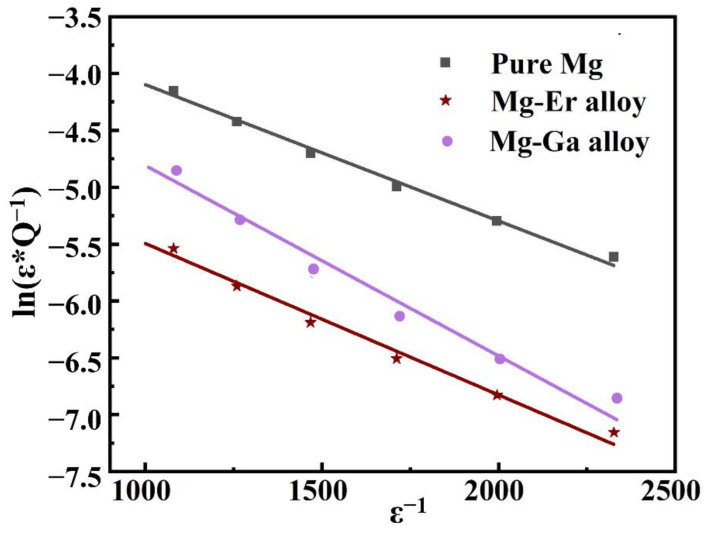
The G-L diagrams of pure Mg, solid solution Mg-Ga and Mg-Er alloys.

Q_H_^−1^ = (C_1_/ε) exp (−C_2_/ε) (3)

(4) $$C_{1}=(\rho F_{B}L_{N}^{3})/(6\mathrm{bE}L_{C}^{2}),\;C_{2}=F_{B/}\mathrm{bE}L_{C}$$

ln(εQ_H_^−1^) = −C_2_/ε + lnC_1_ (5)

where Q_o_^−1^ represents the independent strain component, damping performance is in the low strain stage. Q_H_^−1^ indicates dependent strain component, suggesting its damping performance is in the high strain stage. ε is the strain amplitude, ρ represents the mobile dislocation density, $L_{C}$ denotes the distance between the weak pinning points, $L_{N}$ is the length between the strong pinning points. F_B_ represents the force between point defects and dislocations, and E is the elastic modulus. b indicates the magnitude of Burger’s vector of dislocations. In the high strain stage, ln(εQ_H_^−1^) has a linear relationship with 1/ε. This linear relationship can be used to judge whether the damping mechanism of alloys is dislocation type damping mechanism. As displayed in Figure 6, the G-L diagram of Mg-Ga alloy is obtained by plotting with a range of 1/ε from 1000 to 2500 due to the large critical strain. It can be determined from this figure that the G-L diagram of Mg-Ga alloy is a straight line, indicating that the damping mechanism in Mg-Ga alloy is dislocation type damping. It can be seen from the diagram that the G-L diagram of Mg-Er alloy is a straight line, suggesting that the damping mechanism in Mg-Er alloy is dislocation type damping mechanism. The dislocation damping mechanism can be explained by G-L dislocation pinning theory. In the low strain stage, the movable dislocations in the alloy bow out to and fro between the weak pinning points. The external energy is consumed inside the alloy and the damping value is independent of the strain amplitude. In the high strain stage, the dislocation segment continuously arches out with the constant increase in the strain. When the strain reaches a critical value, the movable dislocations in the magnesium alloy fall off at the weak pinning points but can also be pinned by the strong pinning points. These dislocations that have been unscrewed begin to bow out to and fro between strong stapling points to consume energy. After solution treatment, all the second phases in the alloy disappear, and the stress segregation in the alloy decreases. Through process optimization, the grain size of the alloy is controlled to be similar. The number of strong pinning points in the alloy is reduced and the average dislocation movable length of the movable dislocation line between the strong pinning points is increased. This phenomenon is conducive to the improvement of the damping property of alloys.

## 4. Conclusions

In this work, the effect of solution treatment on the damping capacities of binary Mg-X (X = Ga and Er) alloys was studied. In binary Mg-X (X = Ga and Er) as-cast alloys, alloy elements are dispersed in the magnesium matrix in the form of the second phase. According to the results of XRD and EDS, the specific composition of the second phase (Mg_5_Ga_2_, Mg_24_Er_5_) can be known. After solution treatment, the second phase in the binary Mg-X (X = Ga and Er) solid solution alloys disappears and the alloying elements exist in the matrix of Mg alloys in the form of solid solution atoms. Compared with as-cast alloys, the damping properties of solid solution alloys are improved. The damping mechanism of the two solid solution alloys is G-L mechanism. The radius difference between alloy elements and Mg atom leads to the difference of damping capacities of binary alloys. Solution treatment can reduce the number of the second phase and strong pinning points in the alloy, improving the mobility of dislocations and the damping capacity of the alloys.

## Figures and Tables

**Table 1:** The atomic sizes of elements.

Elements	Atomic Radius (Å)
Ga	1.40
Er	1.76
Mg	1.60

**Table 2:** The damping capacity of four binary Mg-X (X = Ga and Er) alloys and pure Mg.

Alloys	Strain (ε)	Damping Capacity
as-cast Mg-Ga	10^−3^	0.024
as-cast Mg-Er	10^−3^	0.038
solid solution Mg-Ga	10^−3^	0.042
solid solution Mg-Er	10^−3^	0.065
pure Mg	10^−3^	0.180

**Table 3:** Characteristic atomic differences between two alloying element atoms.

Element Atom	Ga	Er
atomic radius (r)	1.40	1.76
atomic radius difference (Δr)	−0.20	0.16
at.%	3.10	6.90
damping capacity	0.042	0.065

Δr: The radius difference between alloy element atoms and Mg atom. at.%: The limit solid solubility of alloy elements.

## Data Availability

Not applicable.
